# Breast cancer treatment-associated cardiovascular toxicity and effects of exercise countermeasures

**DOI:** 10.1186/s40959-016-0011-5

**Published:** 2016-02-25

**Authors:** Anthony F. Yu, Lee W. Jones

**Affiliations:** Department of Medicine, Memorial Sloan Kettering Cancer Center, Cardiology Service, 1275 York Avenue, New York, NY 10065 USA

**Keywords:** Exercise, Breast cancer, Physical activity, Cardiotoxicity, Chemotherapy

## Abstract

Advances in breast cancer treatment have improved disease-free survival and overall survival in women with early-stage breast cancer. However, these improvements may be attenuated by the adverse cardiovascular effects associated with breast cancer adjuvant therapy. Exercise may be a potential strategy to counteract these toxicities. The purpose of this paper is to provide an overview on the adverse cardiovascular effects of breast cancer therapy as well as the evidence supporting the potential cardioprotective effects of exercise training in breast cancer patients during and after treatment. We will also discuss research gaps and avenues for future research.

## Background

Breast cancer is the most common malignancy in women in the United States, with an estimated 231,840 new cases expected to be diagnosed in 2015 [1]. However, earlier detection and advances in adjuvant therapy have resulted in steadily decreasing rates of breast cancer-related death [2]. The 5-year survival rate for breast cancer has improved from 74.6 % in women diagnosed from 1975–1979 to 89.4 % in 2005–2011 [3]. In the U.S. today, there are an estimated 2.8 million survivors of breast cancer, representing 22 % of all cancer survivors [4]. As such, breast cancer patients are living longer and thus are increasingly at risk for the late-occurring adverse effects of cancer therapy. Most notably, breast cancer survivors are at increased risk for cardiovascular disease (CVD) as defined by events such as heart failure and coronary artery disease (CAD). In fact, CVD has now surpassed breast cancer as the leading cause of death among survivors of early-stage breast cancer after 65 years of age [5, 6]. As such, prevention and management of adverse cardiovascular effects is becoming an increasingly important clinical issue in early-stage breast cancer. Current strategies have focused on screening and prevention of cardiomyopathy and include treatment modification based on patient-specific risk profiles, intensive monitoring for early detection of cardiovascular injury using imaging and circulating biomarkers, and pharmacologic intervention with cardioprotective medications (e.g. beta-blockers or ACE-inhibitors) [7].

Exercise, defined as a regular regimen of structured physical activity performed with the goal of improving health or physical fitness [8] is increasingly being recognized as an effective strategy to counter the adverse effects of cancer therapy. The cardiovascular benefit of exercise in the non-cancer setting has been extensively studied, with regular moderate to vigorous-intensity exercise currently recommended by the American Heart Association to reduce cardiovascular risk including lowering of cholesterol and blood pressure [9]. The purpose of this paper is to review the adverse cardiovascular effects associated with breast cancer therapy as well as the available evidence on the efficacy of exercise to mitigate adverse cardiovascular effects in breast cancer patients during and after treatment. We will also discuss gaps in knowledge and avenues for future research.

### Adverse cardiovascular effects associated with breast cancer therapy

Treatment for breast cancer varies depending on multiple tumor-specific factors including size, lymph node involvement, presence or absence of distant metastasis, hormone receptor status, and HER2-receptor status [10]. Taking into consideration these factors, treatment generally utilizes a multimodality approach of surgery, systemic therapy (e.g. chemotherapy, targeted therapy, or endocrine therapy), and radiotherapy. Patient-specific factors and other comorbidities are then taken into consideration in determining the optimal treatment regimen. Here we will review the adverse cardiovascular effects associated with breast cancer therapy.

#### Cardiomyopathy and heart failure

Anthracyclines are a key component of treatment regimens for breast cancer. However, their use is limited by cumulative and dose-dependent cardiotoxicity, which was first reported in the 1970s [11]. Cardiomyopathy and heart failure are the most common clinical manifestation of anthracycline cardiotoxicity. In a review of 4,018 patients treated with doxorubicin, the incidence of doxorubicin-induced heart failure was 2.2 %, and the risk of heart failure increased substantially at cumulative doses of > 400 mg/m^2^ [11]. In a retrospective analysis of 630 patients randomized to doxorubicin plus placebo in three phase III studies by Swain, et al., the percentage of patients with doxorubicin-induced heart failure was 5 at a cumulative dose of 400 mg/m^2^, 26 at a dose of 550 mg/m^2^, and 48 % at a dose of 700 mg/m^2^ [12].

Anthracycline cardiotoxicity can occur acutely within weeks of initial anthracycline exposure, but more commonly presents months to years after treatment. The underlying mechanism of anthracycline-induced myocardial damage has been attributed to multiple adverse effects on cardiomyocytes including oxidative damage from an increase in reactive oxygen species (ROS) formation, alterations in iron homeostasis, and mitochondrial dysfunction [13–15]. These ultimately result in irreversible cardiomyocyte death, and has been classified as type I cancer therapy related cardiac dysfunction (CTRCD) [16]. More recent studies suggest that topoisomerase 2β inhibition may be a key mediator of anthracycline cardiotoxicity [17].

The risk for cardiomyopathy or heart failure is further compounded in patients with HER2-positive disease who receive anthracycline chemotherapy plus anti-HER2 therapy (e.g. trastuzumab). The mechanism of trastuzumab-induced cardiotoxicity is thought to be related to blockade of HER2 signaling that is critical for normal cellular growth and repair mechanisms in cardiomyocytes [18]. In early trials of trastuzumab for metastatic breast cancer, cardiotoxicity was observed in 27 % of patients treated with concurrent anthracycline and trastuzumab therapy, of which 16 % had NYHA class III or IV heart failure [19]. The risk of cardiotoxicity decreased in subsequent adjuvant trials of trastuzumab after several safeguards were implemented, including sequential dosing of trastuzumab following anthracycline-based therapy. In these adjuvant trials the observed rate of heart failure and asymptomatic decline in left ventricular ejection fraction was 0.4–4.1 and 7.1 % - 18.6 %, respectively [20–22].

#### Coronary artery disease

Radiotherapy reduces the risk of recurrence and death from breast cancer, but increases the risk of CAD due to incidental radiation exposure to cardiac structures [23, 24]. Other less common adverse effects associated with radiotherapy include pericardial disease, valvular dysfunction, and conduction abnormalities [25]. In a meta-analysis conducted by the Early Breast Cancer Trialists’ Collaborative Group (EBCTCG) of 40 randomized controlled trials involving 19,582 women, breast cancer mortality was reduced (38.4 % vs. 42.1 %, p-0.0001) but vascular-related mortality was increased (death rate ratio 1.30, *p* = 0.0007) with radiotherapy [26]. Awareness of adverse cardiovascular effects from older radiotherapy regimens from the 1960s and 1970s led to modifications in technique in order to minimize cardiac radiation exposure. In a study by Hooning, et al., the hazard ratio for CVD decreased from 1.49 (95 % CI 1.124 to 1.94) in patients irradiated between 1970–1979 to 1.35 (95 % CI 0.90 to 2.02) in patients irradiated with more modern regimens between 1980–1986 [27].

The extent to which improvements in radiotherapy techniques can reduce harms to the cardiovascular system through dose reduction remains an important clinical question. In a population based case–control study of 2,168 women in Sweden and Denmark with breast cancer treated with radiotherapy between 1958 and 2001, Darby, et al. studied the association between risk of a major coronary event (i.e. myocardial infarction, coronary revascularization, or death from ischemic heart disease) and radiation dose to the heart. The rate of major coronary events increased by 7.4 % for each additional 1Gy of radiation to the heart, and an increase in cardiac risk was present even at low radiation doses ≤ 4 Gy [28]. Of note, results of this study are reflective of older radiation techniques prior to the introduction of newer strategies that further minimize radiation exposure to the heart (e.g. computed tomographic planning or breath-hold technique). Nonetheless, evidence of residual cardiac risk despite a low mean radiation dose to the heart may provide the rationale for newer techniques such as proton beam therapy that can further reduce radiation exposure to cardiac structures, however this requires further investigation.

#### Effects secondary to adjuvant therapy: weight gain and physical inactivity

Beyond the direct insults of systemic therapy and radiotherapy, breast cancer patients are also subject to indirect lifestyle perturbations such as weight gain and physical inactivity, both of which have been associated with adverse cardiovascular events in the non-cancer population [29, 30]. The occurrence of weight gain is common during the first year after breast cancer diagnosis. In a study by Goodwin, et al. of 535 women with newly diagnosed breast cancer, 84 % of patients gained weight after 1 year with a mean weight gain of 1.6 kg [31]. Treatment with chemotherapy was an independent predictor of weight gain, with mean weight increase of 2.5 kg among patients receiving chemotherapy. Physical activity has also been shown to decrease significantly after breast cancer diagnosis. In the Health, Eating, Activity, and Lifestyle (HEAL) study by Irwin, et al., physical activity levels decreased by an estimated 2 h per week in women with breast cancer within 1 year of diagnosis compared to baseline [32]. The largest decrease in physical activity was observed in women treated with radiation and chemotherapy. By 3 years after diagnosis, only 32 % of breast cancer survivors were meeting the recommended levels of physical activity (i.e. 150 min/week of moderate- to vigorous-intensity activity) [33].

#### Cardiorespiratory impairment

Assessment of early and late cardiovascular effects in breast cancer survivors has primarily focused on the evaluation of left ventricular (LV) systolic function. However, breast cancer therapy causes varying degrees of direct (e.g. cardiac dysfunction, pulmonary dysfunction, endothelial dysfunction, skeletal muscle dysfunction) and indirect (e.g. decreased lean body mass, deconditioning) perturbations to the global cardiovascular system that extend beyond the heart. This has been described as the “multiple-hit hypothesis” in which multiple sequential exposures to cardiotoxic treatment are coupled with indirect lifestyle changes and result in overt CVD among breast cancer survivors [10]. Cardiopulmonary exercise testing (CPET) provides an evaluation of exercise capacity (or VO_2peak_), which reflects the ability of the cardiovascular system to deliver oxygen to exercising skeletal muscle and capacity of skeletal muscle to utilize oxygen [34]. Tools such as CPET provide optimal characterization of global cardiovascular impairment related to cancer treatment.

Impaired exercise capacity is becoming a central defining feature in breast cancer patients. In two cross-sectional studies, exercise capacity was significantly lower in breast cancer survivors evaluated several years after completion of therapy compared to age-matched healthy controls [35, 36]. Subsequently, we performed a cross-sectional study in 248 women to evaluate cardiopulmonary function across the breast cancer continuum – before, during, and after adjuvant therapy for nonmetastatic disease, as well as during therapy in the metastatic setting [37]. Despite normal resting left ventricular ejection fraction, women with breast cancer had significant impairments in exercise capacity with mean VO_2peak_ that was 17.8 mL/kg/min or 27 % below age-matched healthy sedentary women. Furthermore, in women with metastatic disease the hazard ratio for death with a VO_2peak_ of > 1.09 L/min was 0.32 (95 % CI, 0.16 to 0.67, *p* = 0.002) compared with a VO_2peak_ ≤ 1.09 L/min, suggesting that VO_2peak_ has prognostic significance. The Alberta moving beyond breast cancer (AMBER) study is an ongoing prospective cohort study that will quantitatively measure physical activity and exercise capacity in 1,500 survivors of early breast cancer [38]. The primary goal of this study is to identify associations between physical activity and exercise capacity on disease outcomes (e.g. breast cancer recurrence and overall survival).

### Cardiovascular benefits of exercise in breast cancer

In adults without cancer, exercise has been associated with many cardiovascular health benefits including reduced risk of ischemic heart disease, stroke, hypertension, dyslipidemia, diabetes, and metabolic syndrome [39–45]. The known cardioprotective effect of exercise has provided a strong rationale to investigate the potential cardiovascular benefit of exercise in the context of breast cancer therapy. Over the last two decades, there has been increasing research interest in the role of exercise to mitigate early and late adverse effects of cancer therapy in breast cancer survivors. Importantly, current evidence has demonstrated the safety and feasibility of exercise during and after cancer treatment. In a meta-analysis of 82 studies on physical activity interventions in cancer survivors (83 % of studies were among women with early breast cancer), Speck, et al. reported that exercise was generally well tolerated and safe with few reported adverse events during or after cancer treatment [46]. In the following sections we will review data from epidemiologic, preclinical, and clinical studies on the cardiovascular benefits of exercise in breast cancer.

#### Epidemiologic studies

Physical activity and weight gain are known predictors of cardiovascular outcomes in non-cancer populations [10]. Furthermore, observational studies have shown that physical activity is associated with reductions in all-cause mortality in women with early-stage breast cancer [47, 48]. However, the prognostic value of physical activity on cardiovascular events in breast cancer survivors has not been well studied. Accordingly, we investigated the association between physical activity and CVD outcomes among 2,973 women with breast cancer in two population-based cohort studies, the Life After Cancer Epidemiology (LACE) and Pathways study. [Jones LW, et al.: Exercise and risk of cardiovascular events in women with non-metastatic breast cancer, submitted]. In this analysis, increasing levels of physical activity were associated with a reduction in cardiovascular events (e.g. coronary artery disease or heart failure) and all-cause mortality. Compared to women engaged in less than 2 MET-hrs/week of leisure-time physical activity, there was a 9 % (HR 0.91, 95 % CI 0.76 to 1.09), 21 % (HR 0.79, 95 % CI 0.66 to 0.96), and 35 % (HR 0.65, 95 % CI 0.53 to 0.80) reduction in the risk of cardiovascular events for women engaged in 2 to 10.9 MET-hrs/week, 11 to 24.5 MET-hours/week, and ≥ 24.5 MET-hrs/week, respectively.

#### Preclinical studies

Animal models investigating the effect of exercise training on cardiovascular toxicity related to breast cancer therapy have largely focused on a model of anthracycline-induced model of cardiomyopathy. Dolinsky, et al. reported that doxorubicin-induced LV remodeling was attenuated by exercise training (i.e. treadmill running) in mice [49]. Similarly, Lien, et al. showed that exercise training prior to doxorubicin treatment attenuated cardiac dysfunction in a rat model of doxorubicin cardiotoxicity [50]. Furthermore, both studies reported that exercise training prevented a decline in expression of sarcoplasmic/endoplasmic reticulum calcium-ATPase (SERCA) 2a, which has been implicated in the pathogenesis of other cardiomyopathies (e.g. ischemic or dilated cardiomyopathy) [51]. Other reported benefits of exercise training in animal models of anthracycline cardiotoxicity include maintenance of normal cardiac mitochondrial function [52], improvement in measures of LV function (e.g. dP/dt_max_ and dP/dt_min_) [53–55], increased expression of glutathione and heat shock protein 72, and decreased expression of p53 [56, 57]. Findings from these preclinical studies suggest that exercise may be an effective strategy to prevent adverse cardiovascular effects of breast cancer therapy and provide a rationale for clinical studies of exercise during and after breast cancer treatment.

#### Clinical studies – during treatment

Several studies have evaluated the effects of exercise during breast cancer therapy for the prevention of adverse CV effects (Table 1). Courneya, et al. randomized 242 patients initiating adjuvant chemotherapy to three groups – usual care, supervised resistance exercise, or supervised aerobic exercise [58]. After a mean duration of 17 ± 4 weeks of exercise training, there was no significant change in patient-reported outcomes of quality of life, fatigue, depression, or anxiety. However, aerobic exercise was able to prevent a decline in VO_2peak_ (25.2 mL/kg/min at baseline to 25.7 mL/kg/min at follow-up) that was observed in the usual care group (24.8 mL/kg/min at baseline to 23.5 mL/kg/min at follow-up). In a follow-up trial, and also the largest randomized controlled trial to date of exercise training during therapy, Courneya, et al. randomized 301 patients to three exercise regimens: standard (25–30 min of aerobic exercise), high (5–60 min of aerobic exercise), or combined high plus resistance exercise. All exercise groups experienced a decline in VO_2peak_ from baseline to completion of chemotherapy, however this decline was partially attenuated in the high exercise group (ΔVO_2peak_ -2.5 mL/kg/min) versus standard or combined groups (ΔVO_2peak_ -3.4 mL/kg/min and −3.6 mL/kg/min, respectively) [59]. Studies to investigate the effect of exercise training during breast cancer treatment on cardiac structure or function are limited. In the only study by Haykowsky, et al., aerobic training did not prevent trastuzumab-induced LV remodeling or decline in left ventricular ejection fraction after 4 months of treatment among women with HER2-positive breast cancer [60].

**Table 1:** Summary of studies investigating the cardiovascular effects of exercise interventions during breast cancer treatment

Author	Patient population	Exercise intervention	Outcome
Courneya et al. 2007 [58]	242 women initiating adjuvant chemotherapy	Supervised aerobic exercise, 15–45 min/d at 60 % - 80 % of VO_2peak_, 3 d/wk (*n* = 78); supervised resistance exercise, 2 sets of 9 exercises at 60 % - 70 % of 1-repetition maximum, 3 d/wk (*n* = 82); or usual care (*n* = 82). All interventions continued for the duration of chemotherapy (mean 17 wks).	↑ VO_2peak_ and ↑ self-esteem with aerobic exercise compared to usual care; ↑ self–esteem, ↑ muscular strength, and ↑ lean body mass with resistance training compared to usual care.
Courneya et al. 2013 [59]	301 women initiating adjuvant chemotherapy	Supervised aerobic exercise, 25–30 min/d at 55 % - 75 % of VO_2peak_, 3 d/wk (STD, *n* = 96); supervised aerobic exercise at 55 % - 75 % of VO_2peak_, 50–60 min/d, 3 d/wk (HIGH, *n* = 101); or combined supervised aerobic exercise (25–30 min/d) and resistance exercise (2 sets of 9 exercises at 60 % - 75 % of 1-repetition maximum), 50–60 min/d, 3 d/wk (COMB, *n* = 104). All interventions continued for the duration of chemotherapy (mean 16 weeks).	Decline in VO_2peak_ attenuated with HIGH compared to COMB. No significant difference between HIGH and STD on VO_2peak_.
Haykowsky et al. 2009 [60]	17 women receiving adjuvant trastuzumab	Supervised aerobic exercise, 30–60 min/d at 60 % - 90 % of VO_2peak_, 3 d/wk × 4 mo.	No change in VO_2peak_, SBP, or DBP; ↑ LVEDV, ↑LVESV, and ↓ LVEF with exercise.
Hornsby et al. 2014 [68]	20 women receiving neoadjuvant doxorubicin plus cyclophosphamide	Supervised aerobic exercise, 15–45 min/d at 60 % - 70 % of VO_2peak_ and planned interval sessions, 3 d/wk × 12 wks (*n* = 10); or usual care (*n* = 10)	↑ VO_2peak_ with exercise
Kim et al. 2006 [69]	41 women undergoing adjuvant chemotherapy	Supervised aerobic exercise, 30 min at 60 % - 70 % of VO_2peak_, 3 d/wk × 8 wks (*n* = 22); or control (*n* = 19)	↓ HR, ↓ SBP, ↑ VO_2peak_ with aerobic exercise compared to control.
MacVicar et al. 1989 [70]	45 women receiving chemotherapy for stage II breast cancer	Supervised aerobic exercise at 60 % - 85 % of maximum heart rate, 3 d/wk × 10 wks (*n* = 18); non-aerobic stretching and flexibility exercises (*n* = 11); or control (*n* = 16)	↑ VO_2peak_ with aerobic exercise compared to non-aerobic stretching and control.
Segal et al. 2001 [71]	123 women with stage I/II breast cancer receiving adjuvant therapy (radiotherapy, hormonal therapy, or chemotherapy)	Self-directed exercise at 50 % - 60 % of VO_2peak_, 5 d/wk × 26 wks (*n* = 40); supervised exercise at 50 % to 60 %% of VO_2peak_ 3 d/wk and self-directed exercise at same level 2 d/wk × 26 wks (*n* = 42); or usual care (*n* = 41).	↑ VO_2peak_ in supervised exercise group compared to control in patients not receiving chemotherapy; no difference between groups in VO_2peak_ among patients receiving chemotherapy.
Vincent et al. 2013 [72]	42 women with stage I-III breast cancer beginning first line adjuvant chemotherapy	Home-based walking program at 50 % - 60 % of maximum heart rate, 3 d/wk × 12 wks.	↑ VO_2peak_ and ↑ 6MWT distance.

#### Clinical studies – after treatment

Exercise has been increasingly recognized for its importance in the rehabilitation of patients after cancer therapy (Table 2) [61]. A randomized controlled trial by Courneya, et al. was performed to determine the effects of exercise training on cardiopulmonary function and quality of life in breast cancer survivors after completion of therapy (e.g. surgery, radiotherapy, and/or chemotherapy) [62]. In this study of 53 patients a median of 14 months after therapy, exercise training consisted of 3 weekly supervised sessions for 15 weeks. VO_2peak_ increased by 0.24 L/min in the exercise group, whereas it decreased by 0.05 L/min in the control group. A similar study by Schneider, et al. included 113 women with a history of breast cancer and evaluated the effect of 2–3 weekly sessions of supervised exercise training for 6 months. Exercise training was associated with improvement in several parameters of cardiopulmonary function including systolic blood pressure, heart rate, and VO_2peak_ [63]. Other beneficial effects of exercise after treatment include improved upper and lower body strength, body weight, body fat percentage, and overall quality of life [46]. No data are available on the effect of exercise training in cancer survivors to mitigate treatment-induced changes in cardiac structure and function.

**Table 2:** Summary of studies investigating the cardiovascular effects of exercise interventions after completion of breast cancer treatment

Author	Patient population	Exercise intervention	Outcome
Courneya et al. 2003 [62]	53 post-menopausal breast cancer survivors after completion of surgery, radiotherapy, and/or chemotherapy	Supervised aerobic exercise, 15 – 35 min/d at 70 % - 75 % of VO_2peak_, 3 d/wk × 15 wks (*n* = 25); or control (*n* = 28).	↑ VO_2peak_ and ↑ self-reported QOL with aerobic exercise compared to control.
Daley et al. 2006 [73]	108 women treated for breast cancer 12 to 36 months previously	Supervised aerobic exercise, 50 min/d at 65 % - 85 % maximum heart rate and RPE 12–13, 3 d/wk × 8 wks (*n* =34); exercise-placebo, 50 min/d of light-intensity body conditioning/stretching, 3 d/wk × 8 wks (*n* = 36); or usual care (*n* = 38).	↑ fitness measured by submaximal walking test with aerobic exercise and exercise-placebo compared to usual care.
Hutnick et al. 2005 [74]	49 survivors of stage I-III breast cancer	Supervised aerobic exercise, 10 – 20 min/d at 60 % - 75 % of VO_2peak_, plus resistance training, total session 40–90 min/d, 3 d/wk × 6 months (*n* = 28); or control (*n* = 21).	↑ VO_2peak_ and ↑ upper body strength
Pinto et al. 2005 [75]	86 women after completing treatment for stage 0-II breast cancer	Home-based aerobic exercise, 10 – 30 min/d at 55 % - 65 % of maximum heart rate, 5 d/wk × 12 wks (*n* = 43); or control (*n* = 43)	↑ fitness (↓ time for 1-mile walk test); no change in BMI or % body fat with aerobic exercise compared to control.
Schneider et al. 2007 [63]	113 women with breast cancer: 96 completed radiation and/or chemotherapy, and 17 undergoing concurrent cancer treatment with exercise	Supervised aerobic exercise, 60 min/d at 40 %–75 % of heart rate reserve, 2–3 d/wk × 6 months	↑ VO_2peak_, ↓ SBP, ↓ resting heart rate, ↓ fatigue with aerobic exercise after completion of cancer treatment.

### Suggested approach to exercise

**Fig. 1:**
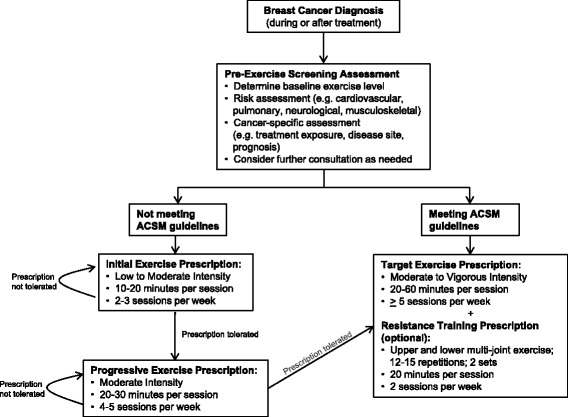
Suggested algorithm for exercise prescriptions in breast cancer patients. A pre-exercise screening assessment should be considered for patients seeking to begin a regular exercise regimen, taking into consideration baseline exercise level, comorbid conditions, and cancer management details. Additional consultation should be considered at the discretion of the treating provider. Patients who do not meet the American College of Sports Medicine (ACSM) guidelines should follow an incremental and step-wise approach to increasing exercise levels. The long-term goal is to perform 20–60 min of moderate to vigorous intensity activity at least 5 days per week. Resistance training can be considered once target exercise levels are achieved. Adapted from references [66, 67], with permission

Based on the growing evidence on the safety and efficacy of exercise training during and after cancer therapy, the American College of Sports Medicine (ACSM) convened an expert panel to formulate exercise guidelines for cancer survivors [64]. These guidelines are consistent with the Physical Activity Guidelines published by the US Department of Health and Human Services [65] and recommend 150 min of moderate-intensity exercise or 75 min of vigorous-intensity exercise per week. For patients diagnosed with cancer, it should be acknowledged that reaching this level of exercise is a long-term goal that will likely require progressive and step-wise increments in frequency, intensity, time, and type of exercise. Furthermore, exercise prescriptions must be individualized on the basis of baseline health status, comorbid conditions, treatment plan, and overall prognosis [66]. A suggested approach to exercise for breast cancer patients (during and after cancer treatment) is outlined in Fig. 1.

### Gaps in knowledge and future directions

Despite the important body of research published to date on the efficacy of exercise training during and after breast cancer treatment, many questions remain unanswered. Overall, data from clinical studies suggest that exercise may attenuate treatment related decline in VO_2peak_ during breast cancer treatment and improve VO_2peak_ after breast cancer treatment. Other benefits are also observed in patient reported outcomes such as quality of life and fatigue. However, the prognostic significance of this improvement remains unknown. Given the increased risk for CVD in breast cancer survivors, the question of whether exercise can improve cardiovascular outcomes is of particular importance. Additional epidemiologic studies utilizing patient cohorts with well characterized data on exercise or physical activity exposure and cardiovascular outcomes are needed to further define the cardiovascular benefit of exercise in breast cancer patients. Due to the latency period between breast cancer treatment and cardiovascular outcomes, prospective studies to answer this question are challenging and will require long-term follow-up. In the absence of this data, studies investigating exercise-induced changes on surrogate measures of cardiovascular structure or function (e.g. imaging or circulating biomarkers) may provide further insight into the cardiovascular benefit of exercise. Other important areas of future research include identifying patient factors that predict response to exercise, and investigating the optimal intensity, timing, and duration of exercise. Finally, the potential for exercise to improve breast cancer outcomes is an exciting new area of research that warrants further investigation.

## Conclusion

Breast cancer treatment has led to significant survival gains but has been associated with adverse cardiovascular effects. Current evidence suggests that exercise may be an effective strategy to mitigate treatment-associated cardiovascular effects in breast cancer patients such as decreased VO_2peak_. Additional studies are needed to further define the effect of exercise on cardiovascular and cancer outcomes.

## References

[1] American Cancer Society. Cancer Facts & Figures 2014. Available at http://www.cancer.org/research/cancerfactsstatistics/cancerfactsfigures2014/. [accessed October 27, 2015].

[2] Siegel R, Naishadham D, Jemal A (2012). Cancer statistics, 2012. CA Cancer J Clin.

[3] Howlander N NA, Krapcho M, Garshell J, Miller D, Altekruse SF, Kosary CL, Yu M, Ruhl J, Tatalovich Z, Mariotto A, Lewis DR, Chen HS, Feuer EJ, Cronin KA (eds). SEER Cancer Statistics Review, 1975–2012, National Cancer INstitute. Bethesda, MD, http://seer.cancer.gov/csr/1975_2012/, based on November 2014 SEER data submission, posted to the SEER website, April 2015.

[4] American Cancer Society. What Are the Key Statistics about Breast Cancer? Available at http://www.cancer.org/cancer/breastcancer/detailedguide/breast-cancer-key-statistics. [accessed January 21, 2015].

[5] Hanrahan EO, Gonzalez-Angulo AM, Giordano SH, Rouzier R, Broglio KR, Hortobagyi GN (2007). Overall survival and cause-specific mortality of patients with stage T1a, bN0M0 breast carcinoma. J Clin Oncol.

[6] Patnaik JL, Byers T, DiGuiseppi C, Dabelea D, Denberg TD (2011). Cardiovascular disease competes with breast cancer as the leading cause of death for older females diagnosed with breast cancer: a retrospective cohort study. Breast Cancer Res.

[7] Khouri MG, Douglas PS, Mackey JR, Martin M, Scott JM, Scherrer-Crosbie M (2012). Cancer therapy-induced cardiac toxicity in early breast cancer: addressing the unresolved issues. Circulation.

[8] Caspersen CJ, Powell KE, Christenson GM (1985). Physical activity, exercise, and physical fitness: definitions and distinctions for health-related research. Public Health Rep.

[9] Eckel RH, Jakicic JM, Ard JD, de Jesus JM, Houston Miller N, Hubbard VS (2014). 2013 AHA/ACC guideline on lifestyle management to reduce cardiovascular risk: a report of the American College of Cardiology/American Heart Association Task Force on Practice Guidelines. J Am Coll Cardiol.

[10] Jones LW, Haykowsky MJ, Swartz JJ, Douglas PS, Mackey JR (2007). Early breast cancer therapy and cardiovascular injury. J Am Coll Cardiol.

[11] Von Hoff DD, Layard MW, Basa P, Davis HL, Von Hoff AL, Rozencweig M (1979). Risk factors for doxorubicin-induced congestive heart failure. Ann Intern Med.

[12] Swain SM, Whaley FS, Ewer MS (2003). Congestive heart failure in patients treated with doxorubicin: a retrospective analysis of three trials. Cancer.

[13] Chen B, Peng X, Pentassuglia L, Lim CC, Sawyer DB (2007). Molecular and cellular mechanisms of anthracycline cardiotoxicity. Cardiovasc Toxicol.

[14] Simunek T, Sterba M, Popelova O, Adamcova M, Hrdina R, Gersl V (2009). Anthracycline-induced cardiotoxicity: overview of studies examining the roles of oxidative stress and free cellular iron. Pharmacol Rep.

[15] Wallace KB (2007). Adriamycin-induced interference with cardiac mitochondrial calcium homeostasis. Cardiovasc Toxicol.

[16] Gianni L, Herman EH, Lipshultz SE, Minotti G, Sarvazyan N, Sawyer DB (2008). Anthracycline cardiotoxicity: from bench to bedside. J Clin Oncol.

[17] Zhang S, Liu X, Bawa-Khalfe T, Lu LS, Lyu YL, Liu LF (2012). Identification of the molecular basis of doxorubicin-induced cardiotoxicity. Nat Med.

[18] Cote GM, Sawyer DB, Chabner BA (2012). ERBB2 inhibition and heart failure. N Engl J Med.

[19] Seidman A, Hudis C, Pierri MK, Shak S, Paton V, Ashby M (2002). Cardiac dysfunction in the trastuzumab clinical trials experience. J Clin Oncol.

[20] Romond EH, Perez EA, Bryant J, Suman VJ, Geyer CE, Davidson NE (2005). Trastuzumab plus adjuvant chemotherapy for operable HER2-positive breast cancer. N Engl J Med.

[21] Piccart-Gebhart MJ, Procter M, Leyland-Jones B, Goldhirsch A, Untch M, Smith I (2005). Trastuzumab after adjuvant chemotherapy in HER2-positive breast cancer. N Engl J Med.

[22] Slamon DJ, Leyland-Jones B, Shak S, Fuchs H, Paton V, Bajamonde A (2001). Use of chemotherapy plus a monoclonal antibody against HER2 for metastatic breast cancer that overexpresses HER2. N Engl J Med.

[23] Clarke M, Collins R, Darby S, Davies C, Elphinstone P, Evans V (2005). Effects of radiotherapy and of differences in the extent of surgery for early breast cancer on local recurrence and 15-year survival: an overview of the randomised trials. Lancet.

[24] Cuzick J, Stewart H, Rutqvist L, Houghton J, Edwards R, Redmond C (1994). Cause-specific mortality in long-term survivors of breast cancer who participated in trials of radiotherapy. J Clin Oncol.

[25] McGale P, Darby SC, Hall P, Adolfsson J, Bengtsson NO, Bennet AM (2011). Incidence of heart disease in 35,000 women treated with radiotherapy for breast cancer in Denmark and Sweden. Radiother Oncol.

[26] Early Breast Cancer Trialists’ Collaborative Group (2000). Favourable and unfavourable effects on long-term survival of radiotherapy for early breast cancer: an overview of the randomised trials. Lancet.

[27] Hooning MJ, Botma A, Aleman BM, Baaijens MH, Bartelink H, Klijn JG (2007). Long-term risk of cardiovascular disease in 10-year survivors of breast cancer. J Natl Cancer Inst.

[28] Darby SC, Ewertz M, McGale P, Bennet AM, Blom-Goldman U, Bronnum D (2013). Risk of ischemic heart disease in women after radiotherapy for breast cancer. N Engl J Med.

[29] Poirier P, Giles TD, Bray GA, Hong Y, Stern JS, Pi-Sunyer FX (2006). Obesity and cardiovascular disease: pathophysiology, evaluation, and effect of weight loss: an update of the 1997 American Heart Association Scientific Statement on Obesity and Heart Disease from the Obesity Committee of the Council on Nutrition, Physical Activity, and Metabolism. Circulation.

[30] Oguma Y, Shinoda-Tagawa T (2004). Physical activity decreases cardiovascular disease risk in women: review and meta-analysis. Am J Prev Med.

[31] Goodwin PJ, Ennis M, Pritchard KI, McCready D, Koo J, Sidlofsky S (1999). Adjuvant treatment and onset of menopause predict weight gain after breast cancer diagnosis. J Clin Oncol.

[32] Irwin ML, Crumley D, McTiernan A, Bernstein L, Baumgartner R, Gilliland FD (2003). Physical activity levels before and after a diagnosis of breast carcinoma: the Health, Eating, Activity, and Lifestyle (HEAL) study. Cancer.

[33] Irwin ML, McTiernan A, Bernstein L, Gilliland FD, Baumgartner R, Baumgartner K (2004). Physical activity levels among breast cancer survivors. Med Sci Sports Exerc.

[34] American Thoracic S (2003). American College of Chest P. ATS/ACCP Statement on cardiopulmonary exercise testing. Am J Respir Crit Care Med.

[35] Jones LW, Haykowsky M, Pituskin EN, Jendzjowsky NG, Tomczak CR, Haennel RG (2007). Cardiovascular reserve and risk profile of postmenopausal women after chemoendocrine therapy for hormone receptor--positive operable breast cancer. Oncologist.

[36] Jones LW, Haykowsky M, Peddle CJ, Joy AA, Pituskin EN, Tkachuk LM (2007). Cardiovascular risk profile of patients with HER2/neu-positive breast cancer treated with anthracycline-taxane-containing adjuvant chemotherapy and/or trastuzumab. Cancer Epidemiol Biomarkers Prev.

[37] Jones LW, Courneya KS, Mackey JR, Muss HB, Pituskin EN, Scott JM (2012). Cardiopulmonary function and age-related decline across the breast cancer survivorship continuum. J Clin Oncol.

[38] Courneya KS, Vallance JK, Culos-Reed SN, McNeely ML, Bell GJ, Mackey JR (2012). The Alberta moving beyond breast cancer (AMBER) cohort study: a prospective study of physical activity and health-related fitness in breast cancer survivors. BMC Cancer.

[39] Gulati M, Pandey DK, Arnsdorf MF, Lauderdale DS, Thisted RA, Wicklund RH (2003). Exercise capacity and the risk of death in women: the St James Women Take Heart Project. Circulation.

[40] Manson JE, Greenland P, LaCroix AZ, Stefanick ML, Mouton CP, Oberman A (2002). Walking compared with vigorous exercise for the prevention of cardiovascular events in women. N Engl J Med.

[41] Nocon M, Hiemann T, Muller-Riemenschneider F, Thalau F, Roll S, Willich SN (2008). Association of physical activity with all-cause and cardiovascular mortality: a systematic review and meta-analysis. Eur J Cardiovasc Prev Rehabil.

[42] Wendel-Vos GC, Schuit AJ, Feskens EJ, Boshuizen HC, Verschuren WM, Saris WH (2004). Physical activity and stroke. A meta-analysis of observational data. Int J Epidemiol.

[43] Whelton SP, Chin A, Xin X, He J (2002). Effect of aerobic exercise on blood pressure: a meta-analysis of randomized, controlled trials. Ann Intern Med.

[44] Helmrich SP, Ragland DR, Leung RW, Paffenbarger RS (1991). Physical activity and reduced occurrence of non-insulin-dependent diabetes mellitus. N Engl J Med.

[45] Warburton DE, Nicol CW, Bredin SS (2006). Health benefits of physical activity: the evidence. CMAJ.

[46] Speck RM, Courneya KS, Masse LC, Duval S, Schmitz KH (2010). An update of controlled physical activity trials in cancer survivors: a systematic review and meta-analysis. J Cancer Surviv.

[47] Holmes MD, Chen WY, Feskanich D, Kroenke CH, Colditz GA (2005). Physical activity and survival after breast cancer diagnosis. JAMA.

[48] Irwin ML, Smith AW, McTiernan A, Ballard-Barbash R, Cronin K, Gilliland FD (2008). Influence of pre- and postdiagnosis physical activity on mortality in breast cancer survivors: the health, eating, activity, and lifestyle study. J Clin Oncol.

[49] Dolinsky VW, Rogan KJ, Sung MM, Zordoky BN, Haykowsky MJ, Young ME (2013). Both aerobic exercise and resveratrol supplementation attenuate doxorubicin-induced cardiac injury in mice. Am J Physiol Endocrinol Metab.

[50] Lien CY, Jensen BT, Hydock DS, Hayward R. Short-term exercise training attenuates acute doxorubicin cardiotoxicity. J Physiol Biochem. 2015. 10.1007/s13105-015-0432-x 26403766

[51] Kawase Y, Ly HQ, Prunier F, Lebeche D, Shi Y, Jin H (2008). Reversal of cardiac dysfunction after long-term expression of SERCA2a by gene transfer in a pre-clinical model of heart failure. J Am Coll Cardiol.

[52] Ascensao A, Lumini-Oliveira J, Machado NG, Ferreira RM, Goncalves IO, Moreira AC (2011). Acute exercise protects against calcium-induced cardiac mitochondrial permeability transition pore opening in doxorubicin-treated rats. Clin Sci.

[53] Hydock DS, Lien CY, Schneider CM, Hayward R (2007). Effects of voluntary wheel running on cardiac function and myosin heavy chain in chemically gonadectomized rats. Am J Physiol Heart Circ Physiol.

[54] Chicco AJ, Schneider CM, Hayward R (2006). Exercise training attenuates acute doxorubicin-induced cardiac dysfunction. J Cardiovasc Pharmacol.

[55] Chicco AJ, Schneider CM, Hayward R (2005). Voluntary exercise protects against acute doxorubicin cardiotoxicity in the isolated perfused rat heart. Am J Physiol Regul Integr Comp Physiol.

[56] Werner C, Hanhoun M, Widmann T, Kazakov A, Semenov A, Poss J (2008). Effects of physical exercise on myocardial telomere-regulating proteins, survival pathways, and apoptosis. J Am Coll Cardiol.

[57] Ascensao A, Magalhaes J, Soares J, Ferreira R, Neuparth M, Marques F (2005). Endurance training attenuates doxorubicin-induced cardiac oxidative damage in mice. Int J Cardiol.

[58] Courneya KS, Segal RJ, Mackey JR, Gelmon K, Reid RD, Friedenreich CM (2007). Effects of aerobic and resistance exercise in breast cancer patients receiving adjuvant chemotherapy: a multicenter randomized controlled trial. J Clin Oncol.

[59] Courneya KS, McKenzie DC, Mackey JR, Gelmon K, Friedenreich CM, Yasui Y (2013). Effects of exercise dose and type during breast cancer chemotherapy: multicenter randomized trial. J Natl Cancer Inst.

[60] Haykowsky MJ, Mackey JR, Thompson RB, Jones LW, Paterson DI (2009). Adjuvant trastuzumab induces ventricular remodeling despite aerobic exercise training. Clin Cancer Res.

[61] Spence RR, Heesch KC, Brown WJ (2010). Exercise and cancer rehabilitation: a systematic review. Cancer Treat Rev.

[62] Courneya KS, Mackey JR, Bell GJ, Jones LW, Field CJ, Fairey AS (2003). Randomized controlled trial of exercise training in postmenopausal breast cancer survivors: cardiopulmonary and quality of life outcomes. J Clin Oncol.

[63] Schneider CM, Hsieh CC, Sprod LK, Carter SD, Hayward R (2007). Effects of supervised exercise training on cardiopulmonary function and fatigue in breast cancer survivors during and after treatment. Cancer.

[64] Schmitz KH, Courneya KS, Matthews C, Demark-Wahnefried W, Galvao DA, Pinto BM (2010). American College of Sports Medicine roundtable on exercise guidelines for cancer survivors. Med Sci Sports Exerc.

[65] Physical Activities Guideliens Advisory Committee (2008). Physical Activity Guidelines Advisory Committee Report.

[66] Jones LW, Eves ND, Peppercorn J (2010). Pre-exercise screening and prescription guidelines for cancer patients. Lancet Oncol.

[67] Metkus TS, Baughman KL, Thompson PD (2010). Exercise prescription and primary prevention of cardiovascular disease. Circulation.

[68] Hornsby WE, Douglas PS, West MJ, Kenjale AA, Lane AR, Schwitzer ER (2014). Safety and efficacy of aerobic training in operable breast cancer patients receiving neoadjuvant chemotherapy: a phase II randomized trial. Acta Oncol.

[69] Kim CJ, Kang DH, Smith BA, Landers KA (2006). Cardiopulmonary responses and adherence to exercise in women newly diagnosed with breast cancer undergoing adjuvant therapy. Cancer Nurs.

[70] MacVicar MG, Winningham ML, Nickel JL (1989). Effects of aerobic interval training on cancer patients’ functional capacity. Nurs Res.

[71] Segal R, Evans W, Johnson D, Smith J, Colletta S, Gayton J (2001). Structured exercise improves physical functioning in women with stages I and II breast cancer: results of a randomized controlled trial. J Clin Oncol.

[72] Vincent F, Labourey JL, Leobon S, Antonini MT, Lavau-Denes S, Tubiana-Mathieu N (2013). Effects of a home-based walking training program on cardiorespiratory fitness in breast cancer patients receiving adjuvant chemotherapy: a pilot study. European journal of physical and rehabilitation medicine.

[73] Daley AJ, Crank H, Saxton JM, Mutrie N, Coleman R, Roalfe A (2007). Randomized trial of exercise therapy in women treated for breast cancer. J Clin Oncol.

[74] Hutnick NA, Williams NI, Kraemer WJ, Orsega-Smith E, Dixon RH, Bleznak AD (2005). Exercise and lymphocyte activation following chemotherapy for breast cancer. Med Sci Sports Exerc.

[75] Pinto BM, Frierson GM, Rabin C, Trunzo JJ, Marcus BH (2005). Home-based physical activity intervention for breast cancer patients. J Clin Oncol.

